# Juvenile Xanthogranuloma Mimicking Other Dermal Nodules: A Diagnostic Challenge in an Infant

**DOI:** 10.7759/cureus.104450

**Published:** 2026-02-28

**Authors:** Rashed A Alhusaini, Alsadat Mosbeh, Abeer Albazali

**Affiliations:** 1 Dermatology, Farwaniya Hospital, Sabah Al Nasser, KWT; 2 Dermatology/Dermatopathology, Faculty of Medicine, Al-Azhar University, Cairo, EGY

**Keywords:** cd68-positive, congenital, juvenile xanthogranuloma, non-langerhans cell histiocytosis, touton giant cells

## Abstract

Juvenile xanthogranuloma (JXG) is a benign histiocytic proliferation that typically presents as solitary or multiple skin lesions in infants and young children. We present the case of a six-month-old female infant who presented with a solitary, firm, dome-shaped yellowish-brown nodule on the back, noticed by her parents four weeks prior to consultation. The lesion was asymptomatic, with no history of trauma or discharge, and was not associated with systemic symptoms. Clinical examination and differential diagnoses included JXG, mastocytoma, Spitz nevus, nevus sebaceous, and xanthoma. A 3 mm punch biopsy was performed, and histological analysis revealed a pandermal infiltrate composed of epithelioid and foamy histiocytes intermingled with Touton-type giant cells, lymphocytes, eosinophils, and plasma cells. The overlying epidermis demonstrated hyperkeratosis and acanthosis, with no evidence of malignancy. The histiocytes were positive for CD68, confirming the diagnosis of JXG. This case underscores the importance of recognizing JXG as a differential diagnosis in pediatric dermatology and highlights the characteristic histological findings that facilitate diagnosis in a clinical context.

## Introduction

Juvenile xanthogranuloma (JXG) is an uncommon skin disorder that is typically harmless and self-resolving. It is marked by the presence of one or more reddish or yellowish papules or nodules on the skin, primarily located on the head, neck, or upper trunk. JXG is classified within the broader category of non-Langerhans cell histiocytoses and predominantly affects young children [1]. This condition involves a proliferation of histiocytic cells that resemble dermal dendrocytes. Diagnosis is based on clinical observations and is confirmed through skin biopsy. Generally, JXG follows a benign trajectory, with spontaneous resolution typically occurring within a few years. While severe cases may affect areas beyond the skin, most commonly the eyes - especially the iris, but also the ciliary body and choroid - which can lead to hyphema, glaucoma, uveitis, or vision loss. Other less common extracutaneous sites that can also be affected are the central nervous system, lungs, liver, and spleen. Although extracutaneous manifestations are rare, they can lead to considerable health issues. Therefore, identifying JXG early is crucial for further assessments to exclude any unnoticed systemic issues. It may be mistaken for other rare conditions such as Spitz nevus, solitary mastocytoma, or, in some cases, nevus sebaceous. Prompt identification is vital since the treatment approaches vary.

## Case presentation

A six-month-old female infant was brought to the dermatology clinic of Farwaniya Hospital by her parents with a solitary skin lesion on the back. The lesion had been noticed by the parents approximately four weeks prior to presentation. It was asymptomatic, non-tender, and had shown minimal increase in size since its appearance. There was no history of trauma, discharge, or similar lesions elsewhere. The infant was otherwise healthy with no systemic symptoms such as fever, weight loss, or visual changes. No family history of similar conditions or lipid metabolism disorders was reported.

**Figure 1:**
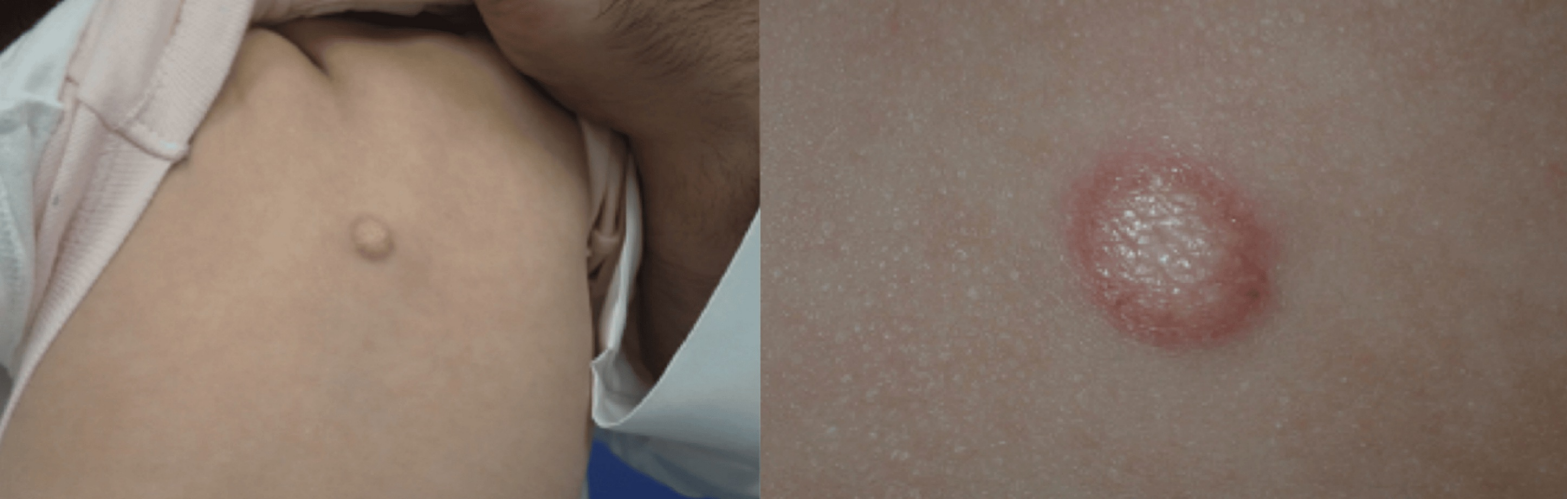
A solitary yellowish-brown nodule on the back of the trunk.

On examination, a solitary, well-defined, firm, dome-shaped, yellowish-brown nodule measuring approximately 1 cm in diameter was seen on the back of the trunk (Figure 1). The overlying skin was intact. No regional lymphadenopathy was noted. The remainder of the systemic examination was unremarkable. Dermoscopy of the lesion revealed a central, yellow-orange, structureless area surrounded by a peripheral erythematous rim, giving a characteristic "setting sun" appearance. Fine linear vessels were observed mainly at the periphery. These dermoscopic features were highly suggestive of JXG, helping to narrow the differential diagnosis. The differential diagnosis included JXG, mastocytoma, Spitz nevus, nevus sebaceous, and xanthoma.

Laboratory investigations, including complete blood count, liver and renal function tests, and lipid profile, were within normal limits. A radiographic examination of the chest and long bones was also performed, showing no evidence of systemic involvement or skeletal abnormalities. A skin punch biopsy (3 mm) from the lesion was done under local anaesthesia. The specimen was bisected and entirely submitted in one cassette.

**Figure 2:**
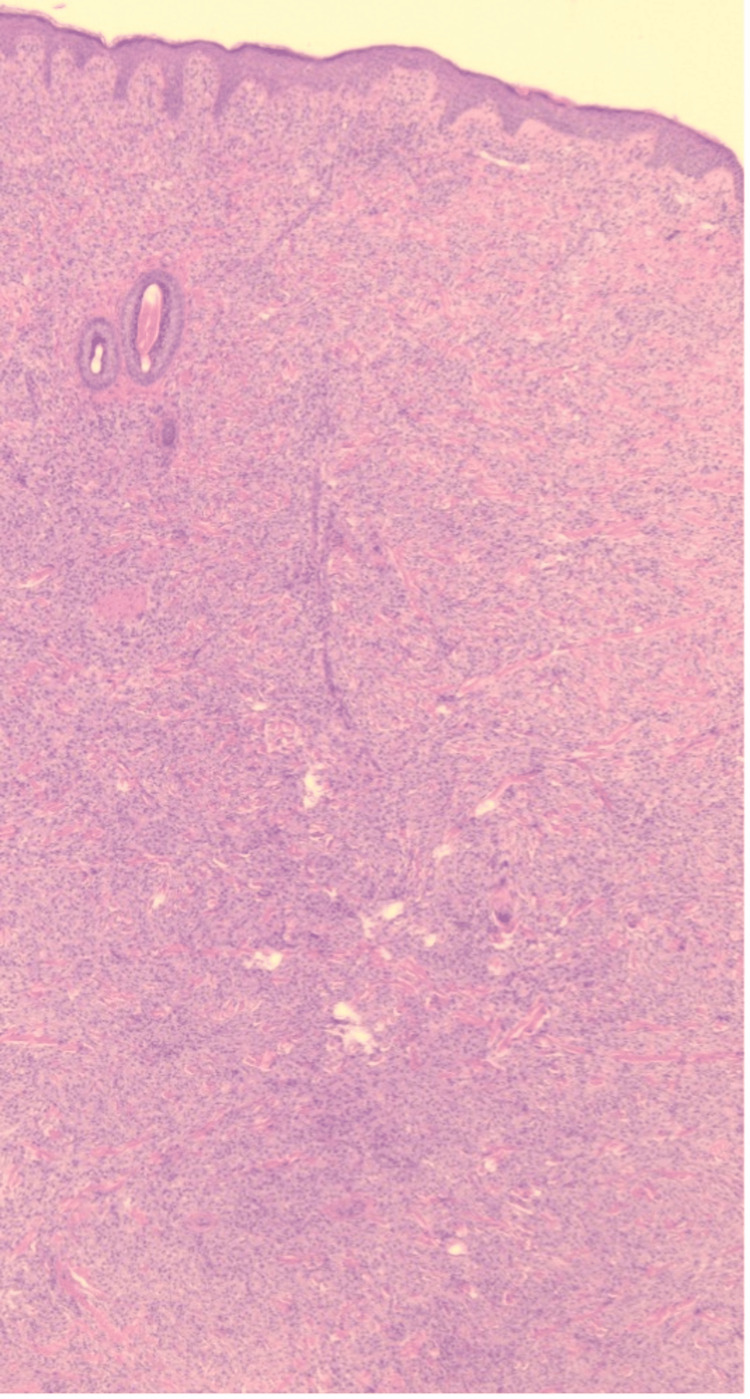
Histopathological image (×4 magnification) showing pandermal infiltration composed of epithelioid and lipid-laden histiocytes, scattered Touton giant cells, and a mixed inflammatory cell infiltrate.

**Figure 3:**
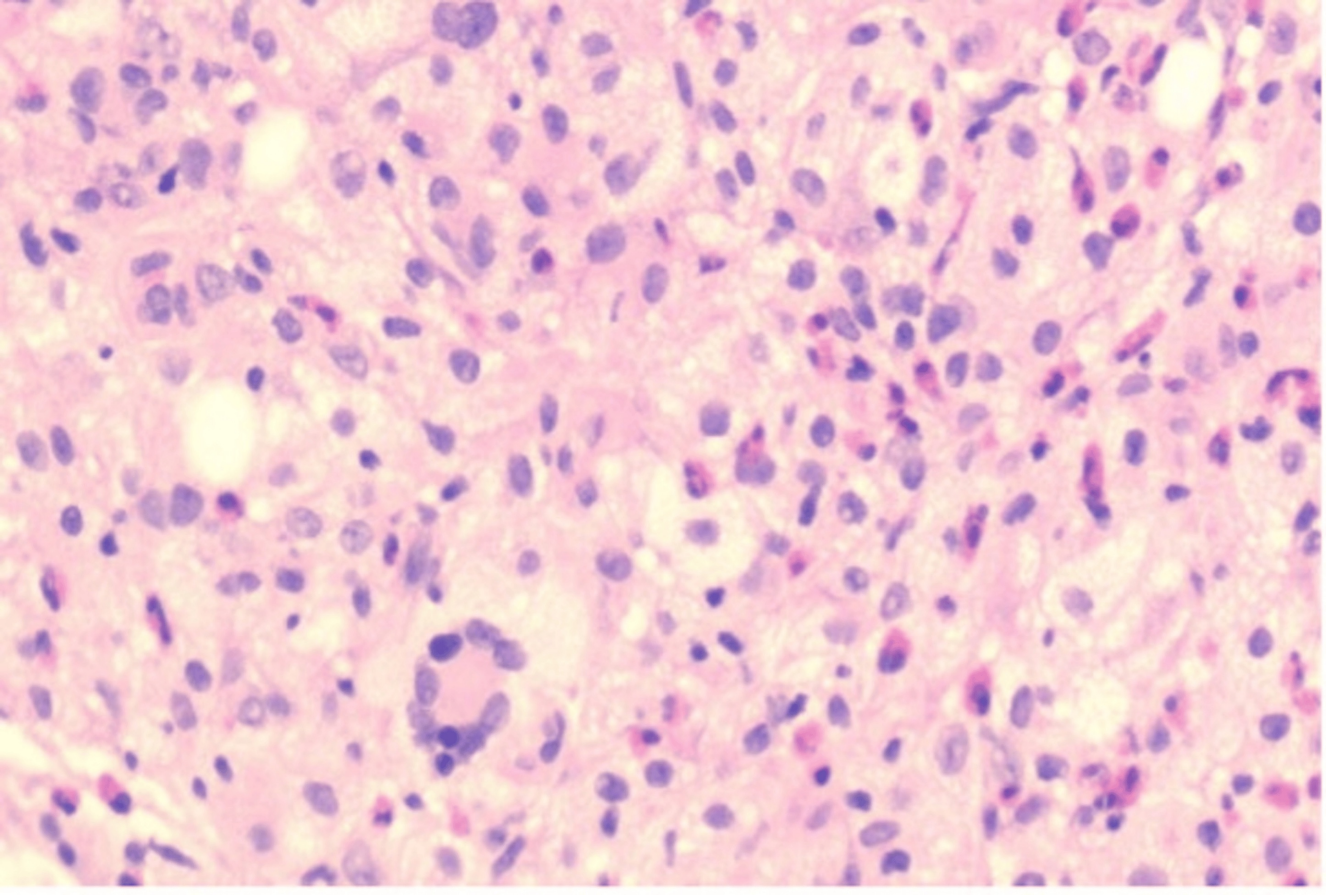
Histopathological image (×20 magnification) showing pandermal infiltration composed of epithelioid histiocytes, foamy histiocytes, Touton-type giant cells, and other inflammatory cells.

**Figure 4:**
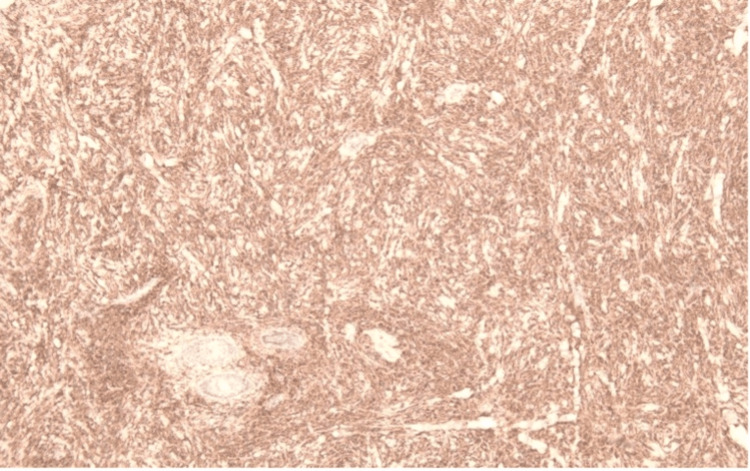
Immunohistochemical staining demonstrating CD68 positivity in histiocytic cells.

Microscopic sections revealed a pandermal infiltrate within the dermis composed of epithelioid and foamy histiocytes admixed with Touton-type giant cells, lymphocytes, eosinophils, and plasma cells. The overlying epidermis shows hyperkeratosis, areas of epidermal thinning alternating with areas of acanthosis with mild elongation of rete ridges. No evidence of atypia, necrosis, or malignancy was identified. Histiocytic cells were positive for CD68. Findings are consistent with JXG (Figures 2-4).

We reassured the parents, as this lesion is self-limiting, and advised follow-up every six months for five years. As the patient had no systemic or ocular involvement, no pharmacologic treatment was initiated, and the patient was managed with reassurance and observation.

## Discussion

JXG is a rare disorder classified under non-Langerhans cell histiocytoses, first identified in 1905 by Adamson as congenital xanthoma multiplex. It is part of a diverse group of histiocytic lesions characterized by a macrophage phenotype and varying reactivity to factor XIIIa and fascin. JXG predominantly occurs in children, typically presenting within the first two years of life [1,2]. The current case is a six-month-old female.

The estimated prevalence is approximately one case per million children. Adult-onset JXG accounts for only about 10% of all cases. A large tumor registry found JXG in 129 out of 24,600 pediatric tumors studied over 35 years [3]. While boys are more frequently affected than girls, no significant differences in prevalence rates have been noted for adult-onset cases [4]. Additionally, pediatric JXG shows a low occurrence of ocular and systemic manifestations [5]. Our case was free from ocular and systemic manifestations.

The exact causes and mechanisms underlying JXG remain unclear. It is believed that the condition arises from an overproduction of a specific type of histiocyte, which plays a role in the immune response to nonspecific tissue damage. JXG typically presents as a well-defined papule, plaque, or nodule that ranges from reddish to yellowish-orange in color, with sizes varying from 0.5 to 2 cm in diameter, and features a smooth surface and firm texture [6-13]. While lesions can occur anywhere on the body, they are most commonly found on the head, neck, and upper trunk [13]. The present case was located on the upper back as a solitary 1-cm yellowish-brown nodule.

Early-stage lesions are often more elevated and reddish, while mature lesions tend to become flatter and acquire a yellowish hue due to lipid accumulation. Although JXG lesions are generally solitary, they can also appear in multiple forms and may involve extracutaneous and systemic sites. Affected organs can include the liver, lungs, spleen, lymph nodes, bones, and gastrointestinal tract [2]. Cases with systemic involvement are often associated with frequent activating mutations in genes related to the mitogen-activated protein kinase pathway [7]. In our case, there was no systemic involvement.

To confirm the clinical diagnosis of JXG, a skin biopsy is necessary. The classic histological features of JXG include a thick, well-defined, non-encapsulated lymphohistiocytic infiltrate composed of mononuclear cells, multinucleated giant cells with or without Touton-type characteristics, and spindle cells extending into the dermis and subcutaneous fat [8]. Although the epidermis may shrink and, in rare cases, become ulcerated, the adnexa and epidermis remain untouched. Touton's gigantic cells are distinguished by a ring of eosinophilic to foamy vacuolated cytoplasm with a high lipid content and a centre wreath of nuclei.

The stage of the lesion affects the microscopic appearance. In the early stages of JXG, only histiocytes, spindle-shaped fibrohistiocytic cells with lipid-free macrophages, are seen. In the superficial dermis, more mature JXG is characterized by lipid-laden mononuclear cells, vacuolated foamy macrophages, and Touton multinucleated giant cells [4,8]. Immunostaining is essential for diagnosis confirmation. JXG is positive for fascin, CD68, CD163, CD14, and factor XIIIa, an interstitial dendrocyte marker. S100 and CD1a staining, the latter of which is exclusive to Langerhans cells, typically yield negative results [8]. The "setting sun pattern," which is a pale lesion with an erythematous halo encircled by peripheral linear telangiectasias in mature JXG, was discovered via dermoscopy [4]. The current case shows typical histologic features of xanthogranuloma and confirmed by positive CD68.

The site of involvement determines the prognosis and course of treatment for JXG. Typically, solitary lesions are benign, self-limiting, and disappear over a period of five years. Ocular JXG is often isolated from cutaneous involvement and primarily affects the iris. Consequently, routine referral of patients under the age of two to an ophthalmologist is not recommended unless they present with multiple micronodular (10 mm) JXG lesions [5]. The advantages of routine ophthalmologic examination for children with cutaneous JXG have not been the subject of any prospective trials. Conversely, the clinical history of the patient may be used to guide diagnostic testing.

To rule out visceral-systemic involvement or the possibility of additional benign tumors, all children with numerous cutaneous JXG lesions, especially those under two years old, should be examined for systemic JXG. This screening should include a brain, chest, and belly CT or MRI [9-11]. Hepatic failure, severe hypercalcemia, progressive central nervous system involvement, and myeloid leukemia may complicate systemic cases in which histiocytes infiltrate the viscera, thereby increasing morbidity and mortality [12]. Low-dose chemotherapy is required for systemic JXG since it affects sensitive areas like the brain or larynx [4]. We reassured the parents that this lesion is a self-limiting one, and we advised them to follow up every six months for five years.

## Conclusions

Most people with JXG have benign, self-limiting symptoms that usually start in childhood and have an unclear cause. Despite being a clinical diagnosis, further testing should be performed by a specialist to rule out systemic involvement. Although extremely uncommon, extracutaneous or systemic symptoms might cause significant morbidity in certain patients. The most common and susceptible extracutaneous sites include the liver, spleen, lung, and central nervous system. A thorough and comprehensive assessment is necessary to prevent disease complications and mortality, even though systemic involvement is not very common.
